# Explicit and implicit sense of agency in depersonalisation experiences

**DOI:** 10.1038/s41598-024-65862-z

**Published:** 2024-07-04

**Authors:** Anna Ciaunica, Julia Ayache, Patrick Haggard, Estelle Nakul, Emmanuelle Bonnet, Malika Auvray

**Affiliations:** 1 Centre for Philosophy of Science, University of Lisbon, Campo Grande, 1749-016 Lisbon, Portugal; 2 Institute of Cognitive Neuroscience, University College London, London, WC1N 3AZ UK; 3 NTU Psychology, Nottingham Trent University, Nottingham, UK; 4 EuroMov Digital Health in Motion, University Montpellier IMT Mines Alès, Montpellier, France; 5 Laboratory of Cognitive Neuroscience (LNC), FR3C, CNRS, Aix Marseille University, Marseille, France; 6 Institut de Neurosciences de la Timone (INT), CNRS, Aix Marseille University, Marseille, France; 7 Institut des Systèmes Intelligents et de Robotique, ISIR, CNRS, Sorbonne Université, 4 Place Jussieu, 75005 Paris, France

**Keywords:** Depersonalisation, Agency, Intentional binding, Voluntary actions, Time perception, Psychology, Human behaviour

## Abstract

The sense of agency, the feeling of controlling one’s bodily actions and the world is altered in Depersonalisation (DP), a condition that makes people feel detached from one’s self and body. To investigate the link between depersonalisation and both implicit and explicit sense of agency, an online study was conducted using the influential Intentional Binding paradigm in a sample of non-clinical DP participants. The results did not reveal significant differences between individuals with low and high occurrences of DP experiences on the implicit and explicit sense of agency. However, participants with high occurrences of DP experiences showed a more time-sensitive explicit sense of agency and greater temporal distortions for short intervals in the absence of self-initiated motion. These results suggest that there is a discrepancy between implicit and explicit sense of agency in people with high levels of depersonalisation. Altogether, these findings call for further investigations of the key role of time perception on altered sense of self and agency in both non-clinical and clinical populations, to disentangle the mechanisms associated with the explicit and implicit sense of agency.

## Introduction

The sense of agency refers to the subjective feeling of voluntarily controlling one’s bodily actions, and through them events in the outside world^1,2^. Depersonalisation (DP henceforth) is a common condition characterised by feelings of being detached from one’s self, body, and the world^3^ and ‘watching’ oneself acting in the world from a distant viewpoint: “*When I’m having an episode of depersonalisation, it feels more like I’m watching myself doing things, but I’m not present for it. I’m witnessing myself… I ‘know’ I’m in control, but I’m not ‘feeling’ in control*”^4^.

First described by Théodule Ribot as “folie du doute”, the condition was extensively studied by Dugas^5^ who coined the term “depersonalisation” to refer to a failure of “personalisation”, i.e. a lack of “attachment” to the self^3^. DP is also characterised by feelings of “unrealness”^6^ or doubt about what is real or not: “I find myself regarding existence as though from beyond the tomb, from another world. (…) I am as it were, outside my own body and individuality”^7^. Emotional numbing (also coined as apathetic insanity^5^) and disruption of autobiographical memory^8^ are also key aspects of DP experiences^9^. As Hesnard^10^ noted, these disturbances lead individuals suffering from DP to develop an excess of “internal attention” characterised by an acute introspection as a mechanism to retrieve and grasp the “lost” self.

More recently, four main experiential factors have been identified as fundamentally related to DP:

(a) anomalous feelings of “disembodiment” and “estrangement” from one’s bodily experiences; (b) emotional numbing; (c) anomalous subjective recall; (d) alienation from surroundings (i.e., derealisation)^11^. These symptoms are present cross-culturally^12–17^. DP has a prevalence of around 1–2% of the population and is very common in the young population, with almost 50% of college students reporting having experienced DP^12–17^. Everyday phenomena such as fatigue^18^, sleep deprivation^19^, recreational drugs or travelling to new places may also trigger transient depersonalisation feelings—see^20,21^ for recent discussions. While these symptoms can become chronic, leading to Depersonalisation Disorder (DSM-5^22^), the present study focus on non-clinical experiences of DP.

Anomalous bodily experiences and atypical sense of agency are some of the most commonly reported DP symptoms^11,23^. For example, Sierra et al.^11^ indicated that people diagnosed with DP frequently complain about feelings of not being in control of their bodily movements accompanied by changes in their experience of time and space, as well as perceptual anomalies, such as perceiving the world as being ‘unreal’.

Scientific investigations of the sense of agency have important roots in the study of mental chronometry, investigating the temporal discrepancy between an action and its perception—see^24^ for a historical perspective and^25^ for a recent review. These paradigms were subsequently used to develop implicit measures of the sense of agency based on temporal estimation^26–34^. These implicit measures are often preferred to explicit judgements of agency in experimental settings. Indeed, explicit judgements of agency are rare in everyday life, and known to be influenced by additional factors, such as perceived self-efficacy^35^.

The intentional binding (IB henceforth) paradigm has been proposed as an influential implicit measure of the sense of agency. IB refers to the phenomenon of perceived temporal compression between a voluntary action and its subsequent consequence^36^. The delay between a voluntary action followed by a sound is be perceived as shorter than a comparable delay between a passive movement and the same sound, or between two externally generated sounds^24,29,36^. While the IB paradigm is widely used as an implicit measure of the sense of agency, the mechanisms subserving IB are still under discussion^30,37^ with studies suggesting that dopaminergic processes may play a key role in boosting action-effect association^24,38,39^. Moreover, several authors raised the question of whether the intentional binding effect truly requires an ‘intention’^40–42^. Hence it has been proposed to refer to the phenomenon as “temporal binding”^33^. However, a concept of intentional binding seems appropriate to characterize the difference between a condition that involves intentional action and one that does not.

A recent meta-analysis suggested that altered sense of agency correlates with symptomatology severity and hence provide an important marker for neuropsychiatric disorders^43^. Altered sense of agency is consistently reported as one of the most common characteristics of self-disorders such as schizophrenia and psychosis^44,45^. For example, Haggard and colleagues^45^ reported that the perceived interval between a voluntary action and its consequence appears to be shorter for patients than for controls. This result suggests that patients may overassociate their actions with subsequent events. However, this pattern varies across the schizophrenia symptomatology spectrum^46^ and it remains to be clarified if this altered sense of agency is associated with abnormal temporal perception (i.e., temporal binding^47–49^ or impairments in meta-cognitive processes^50^. Despite plausible evidence showing an association between depersonalisation and self-reports of disrupted sense of agency^51^, to date DP phenomena have not yet been investigated in relation to IB. This is surprising because there is a significant overlap in symptomatology between psychosis and depersonalisation^52^.

To explore the link between DP and the sense of agency in non-clinical population, an online study was conducted, combining the intentional binding task as an *implicit* measure of the sense of agency^36^ with self-reports measuring the *explicit* sense of agency^53^. Although explicit and implicit measures are widely used in the literature, it has been argued that they are driven by different mechanisms, hence it is important to examine them in tandem^28,54^. Previous studies showed an association between IB and the feelings of control^26,55^, suggesting an association between implicit and explicit measures of agency in non-clinical population^53^. However, these measures display discrepancies in clinical populations^56^, hence it is important to explore their associations in DP. Finally, given that IB critically depends on time estimation, our study also explored the link between self-reports of DP experiences, measured by the Cambridge Depersonalisation Scale (CDS) and distorted experiences of self, space and time, measured by the Self, Space and Time” Questionnaire (SST).

Considering that DP is commonly linked to feelings of agency loss^3^ and to feelings of performing actions on ‘automatic pilot’^4^, we hypothesised that participants with higher occurrences of DP experiences (CDS scores > 50) will show lower sense of explicit agency compared to participants with lower occurrences of DP experiences (CDS scores < 20). Furthermore, mirroring their alterations of explicit sense of agency^3^ we hypothesised that the explicit subjective experience of altered sense of agency in DP will affect the implicit sense of agency as measured with the IB effect. In particular, we expected participants with higher occurrences of DP experiences to display a “lower” intentional binding effect. Finally, we conducted exploratory analyses of the link between subjective time perception, DP traits, and IB task performance, as time perception is a fundamental feature of human sense of self and presence in the world. We expected participants differing in DP traits to also differ in subjective time perception and IB task performance.

## Methods

### Participants

868 participants (mean age = 31.32 ± 8.78, 386 men, 472 women, 10 non-binary) were first prescreened by completing a French version^57^ of the Cambridge Depersonalisation Scale (CDS-29 ^3^,). Participants were recruited online from May 2022 until June 2023 via Aix Marseille University email listings, SONA system at University College London, UK and social media using snowball sampling. Inclusion criteria listed: (i) between 18 and 60 of age; (ii) fluent in French; (iii) no history of neurological illnesses; (iv) lack of uncorrected auditory or visual impairments, and (v) lack of drugs consumption during the last 6 months.

From these, 115 eligible participants performed the IB task, with a subsample of 57 HIGH DP (CDS-29 scores > 50) and 58 LOW DP (CDS-29 scores < 20). 22 participants were excluded due to missing values (*N* = 3), an excessive number of missed trials (> 15%, *N* = 13), and for outlier values of temporal estimations (> 2 SD, *N* = 6). Consequently, our final sample consisted of 93 participants divided in HIGH DP (*N* = 46, mean age = 26.91 ± 5.76 years, 5 men, 41 women) and LOW DP (*N* = 47, mean age = 33.89 ± 8.14 years, 25 men, 22 women). A sensitivity power analysis confirmed that this sample size was sufficient to detect a small effect size (f2 = 0.03). Participants were compensated with a €10 voucher for their participation in the IB task. Informed consent was obtained from all participants before the start of the experiment according to procedures approved by the ethics committee from Aix Marseille University (no 2021–09-07–13). The experiment was conducted in accordance with the Declaration of Helsinki.

### Questionnaires

#### Cambridge depersonalisation scale (CDS-29)

CDS-29 is a 29-items standard questionnaire used to evaluate the severity of occurrence of depersonalisation experiences by asking participants to estimate their frequency and duration in the past six months. The total score (between 0 and 290) points is calculated by summing over all items. CDS-29 has good statistical properties^12–17^ with internal reliability for different language versions reported between 0.89 and 0.94 (Cronbach alpha). Moreover, previous research extracted four subscales from CDS-29^11^: (i) Anomalous Body Experience, (ii) Emotional Numbing, (iii) Anomalous Subjective Recall, and (iv) Alienation from Surroundings*.* An investigation into the psychometric properties of our CDS-29 scores revealed that internal reliability was similarly high to previous studies, with Cronbach’s alpha = 0.95*.*

The average total score for the CDS-29 in our sample was 88.89 with median at 81 points, mode at 7 points, and range between 0 and 243 points (with theoretical maximum being 290 points). The distribution of scores was slightly right skewed (Skewness = 0.01) and slightly leptokurtic (Kurtosis = 1.68) reflecting the fact that the majority of participants reported scores between 43 and 141.

#### Self, space and time” questionnaire (SST)

Additionally, all participants completed the “Self, Space and Time” Questionnaire (SST) composed of 14 items, scored on 5-point Likert scales (see Supplementary Material-I). The SST was developed by one of us (AC) to explore in depth the link between DP and altered spatiotemporal perception^58^ in relation to the bodily self. Non-parametric test comparisons revealed significant differences between HIGH DP and LOW DP participants for all the SST items, except the items 2 and 4 (see Table D in Supplementary Material).

### Procedure and stimuli

The experiment was conducted online, on participants’ home computers. Participants provided their informed consent and they first completed a (i) standard demographic questionnaire (age, gender), (ii) the CDS and (iii) the SST questionnaires online through Qualtrics and Pavolvia software^59^. Eligible participants scoring higher than 50 points on CDS-29 (HIGH DP group) and lower than 20 points on CDS-29 (LOW DP group) were invited via email to complete the IB task on Pavolvia. Participants were instructed to use headphones to ensure good sound quality and to find a quiet place to avoid being distracted during the 30 min of the experiment.

For the IB task, an adapted version, combining implicit and explicit measures of agency^53^ was used. Following the classic IB design, two conditions were implemented: (i) **BASELINE** condition, in which participants estimated the delay between two sounds; and (ii) **OPERANT** condition, in which participants estimated the delay between a self-initiated action (i.e., pressing “space” on the keyboard) and a sound. Before each condition, a practice session was included, consisting in 22 trials to familiarize participants with the task instructions.

**Figure 1:**
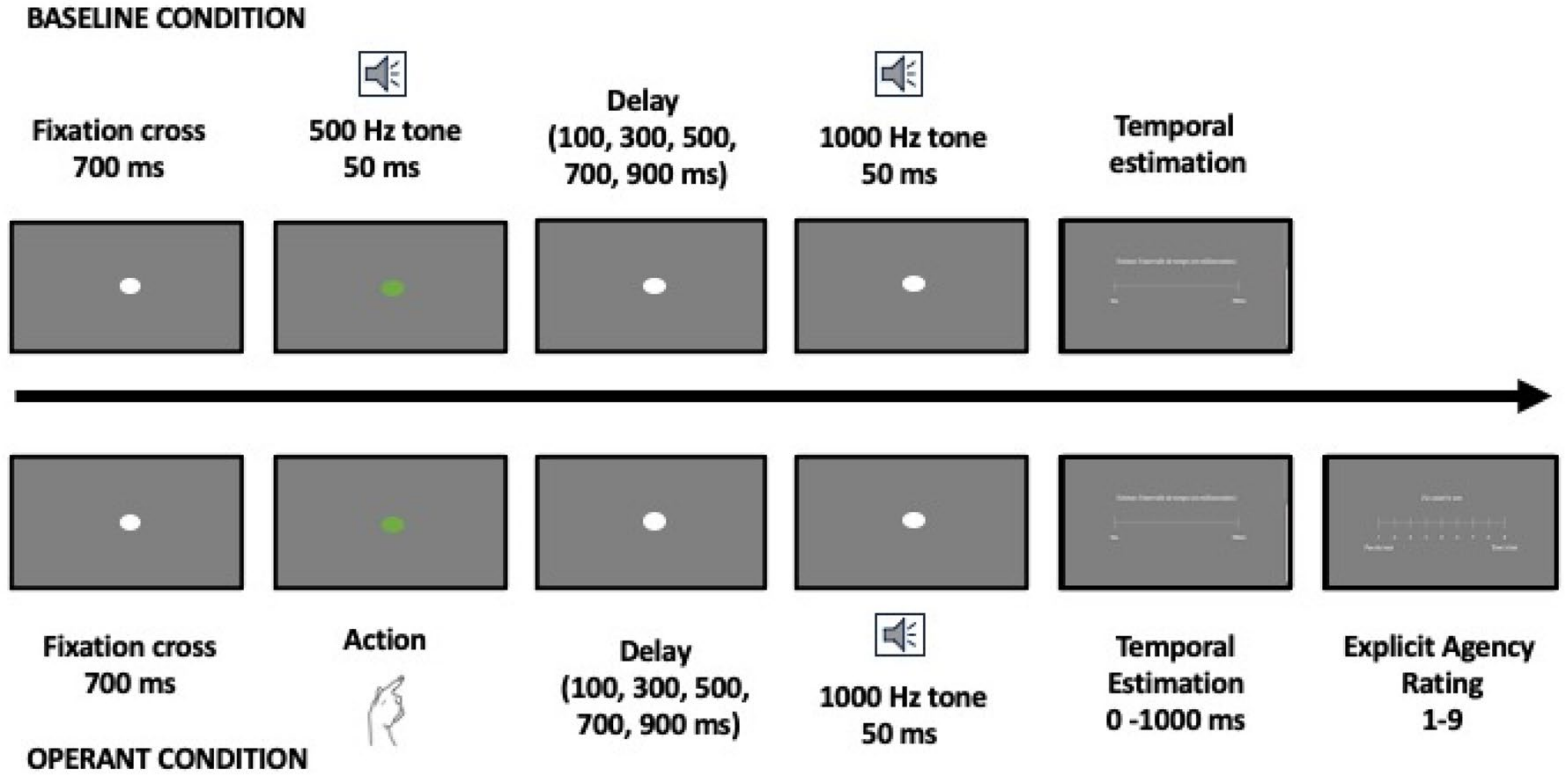
Graphical representation of the intentional binding task, with the **BASELINE** (top raw) and the **OPERANT** (bottom raw) conditions.

Auditory stimuli consisted in two pure tones of 500 and 1000 Hz presented for a duration of 50 ms at a comfortable volume for the participants. A white dot was presented at the center of the screen as a fixation point that turned green after 500 ms as a start signal for performing the action in the **OPERANT** condition. Participants were instructed to press the button as soon as the fixation point turned green. In both conditions, the delays between the two sounds (**BASELINE**) and action-sound (**OPERANT**) were manipulated randomly with values ranging from 100 to 900 ms in increments of 200 ms with an inter-trial interval of 700 ms. Five interval durations (100, 300, 500, 700, 900) were presented 15 times each, resulting in 75 trials for each condition (150 trials in total), with a 1-min break after every 33rd trial (2 breaks in total). For both conditions, participants reported the delay between the two sounds and action-outcome using a visual analog scale ranging from 0 to 1000 ms. Additionally, in the **OPERANT** condition, participants completed a 9-point Likert scale for agency rating. To ensure the effective completion of the task, 15 “catch trials” were also implemented, with participants instructed to press the letter “c” on their keyboard when a cicada sound was presented 150 ms after the first stimulation onset. The completion of the intentional binding task took around 30 min (see Fig. 1 for a graphical representation of the task).

### Data analyses

Data were pre-processed using Matlab and R^60,61^ and several indices were extracted:

The *explicit sense of agency* was calculated for each participant by (i) averaging the 9-point Likert scale scores for each interval duration and (ii) by extracting coefficient slopes of the linear regressions of the interval duration on the explicit sense of agency*.*

The *implicit sense of agency* was estimated from (i) the averaged temporal estimation for each condition (i.e., **BASELINE** versus **OPERANT**) and interval duration, (ii) the temporal estimation difference between the **BASELINE** and **OPERANT** conditions (BASELINE–OPERANT conditions) for each interval duration (i.e., intentional binding) and from (iii) the coefficient slopes of the linear regressions of the interval duration on intentional binding.

For the *explicit sense of agency*, mixed models were computed testing (i) the interaction of interval durations (i.e., 100, 300, 500, 700 and 900) and DP groups (i.e., **HIGH**, **LOW**) as fixed predictors on explicit agency and (ii) the association of DP groups as fixed predictors of overall explicit agency scores (i.e., coefficient slopes).

For the *implicit sense of agency*, mixed models were computed testing (i) the interaction of interval durations, experimental conditions (i.e., BASELINE, OPERANT) and DP groups as fixed predictors of temporal estimation, (ii) the interaction of interval durations and DP groups as fixed predictors of intentional binding and (iii) the main effect of DP groups as fixed predictors of overall intentional binding (i.e., coefficient slopes).

Participant IDs were added as random intercept and post hoc comparisons were performed using Holm Bonferroni corrections for multiple comparisons. Group comparisons across **HIGH** DP and **LOW** DP participants revealed significant differences between **HIGH** and **LOW** groups for age *t* (91) =  − 4.78, *p* < 0.001, 95% CI [− 9.88; − 4.08] and gender (*p* < 0 0.001). Therefore, these variables were added as fixed predictors to control for potential confound.

Pearson’s correlations were calculated between the coefficient slopes of explicit sense of agency and intentional binding. The role of CDS facets was investigated by adding CDS facets as fixed predictor of coefficient slopes of implicit and explicit sense of agency. These analyses were restricted to the **HIGH** DP group due to skewed distribution in the **LOW** DP group. Finally, non-parametric Spearman’s correlations were performed for investigating the association of coefficient slopes of implicit and explicit sense of agency with SST items.

## Results

### Explicit sense of agency

**Figure 2:**
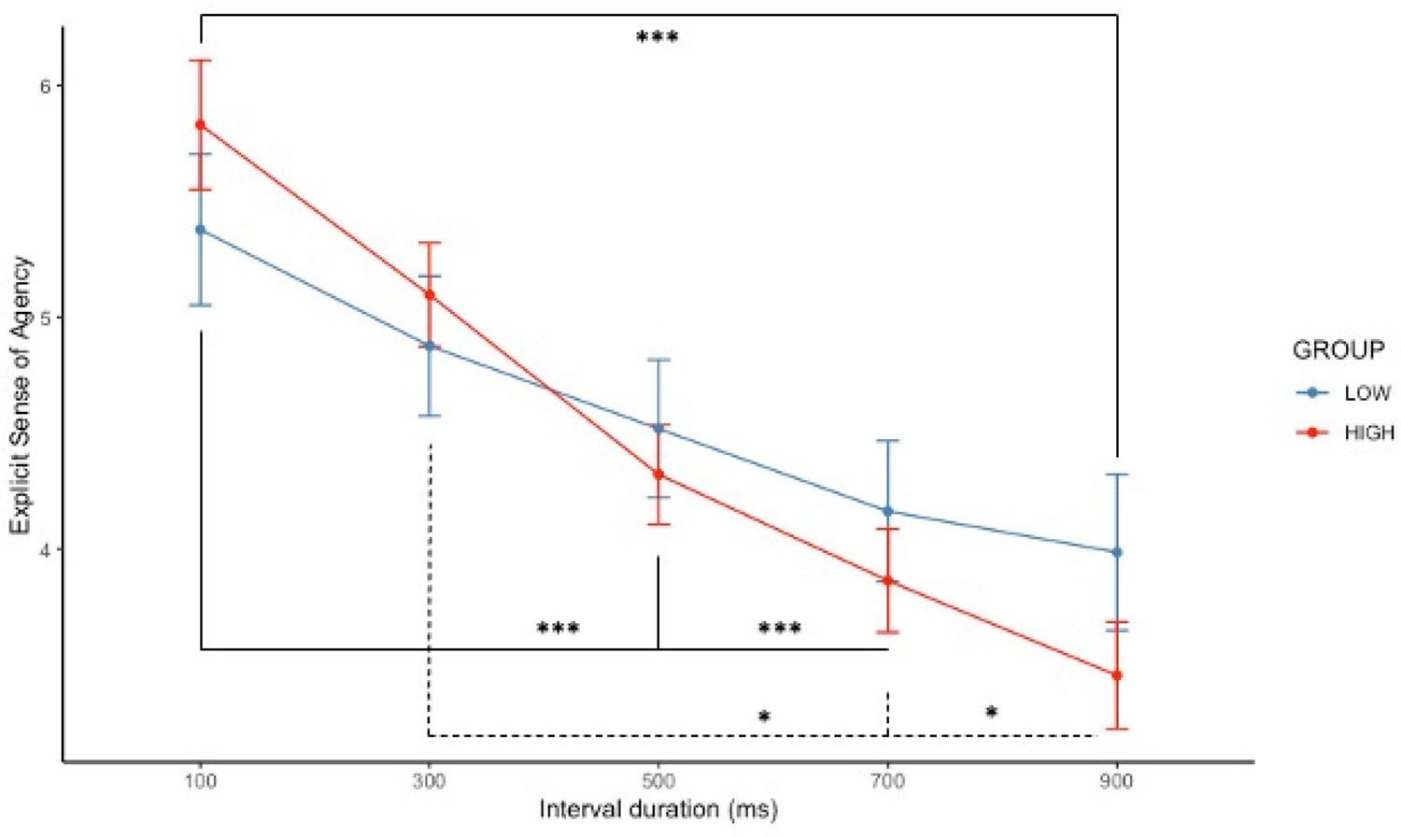
Graphical plot of explicit sense of agency for **LOW** (blue) and **HIGH** (red) DP. The bars represent standard errors and *p-*values are reported with *p* < 0.050*, *p* < 0.010** and *p* < 0.001***.

A significant main effect of interval durations was observed on explicit sense of agency (*F* (4, 364) = 48.51, *p* < 0.001 and *R*^*2*^ = 0.73). Additionally, an interaction was observed between interval durations and DP groups (*F* (4, 364) = 3.39, *p* = 0.010). Post-hoc comparisons revealed that explicit sense of agency was significantly higher for the 100 ms compared to the 500 (*beta* =  − 0.86, *p* < 0.001, 95% CI [− 1.28; − 0.44]), 700 (*beta* =  − 1.22, *p* < 0.001, 95% CI [− 1.64; − 0.79]), and 900 ms interval durations (*beta* =  − 1.39, *p* < 0.001, 95% CI [− 1.82; − 0.97]) and for the 300 ms compared to the 700 and 900 ms interval durations (both *ps* < 0.050). However, there was only a significant difference between the 100 and the 900 ms (*p* = 0.020) in the **LOW** DP group. Despite a trend for participants with **HIGH** CDS scores (mean =  − 3.00 ± 2.59) to display a steeper coefficient slope compared to participants with **LOW** CDS scores (mean =  − 1.75 ± 3.39), there was no significant difference between **HIGH** and **LOW** DP groups (*F* (1, 89) = 3.90, *beta* = 1.05, *p* = 0.189 and *R*^*2*^ = 0.83). See Fig. 2 for a graphical representation and Table A in supplementary material for descriptive statistics.

### Implicit sense of agency

A significant main effect of interval durations was observed on temporal estimations (*F* (4, 819) = 1153.42, *p* < 0.001 and *R*^2^ = 0.84). Post-hoc comparisons showed that participants reported significantly longer temporal estimations for 300 (*beta* = 156.13, *p* < 0.001, 95% CI [122.30; 189.97]), 500 (*beta* = 333.44, *p* < 0.001, 95% CI [299.60; 367.27]), 700 (*beta* = 476.10, *p* < 0.001, 95% CI [442.26; 509.94]) and 900 ms interval durations (*beta* = 591.99, *p* < 0.001, 95% CI [558.15; 625.82]). There was also a main effect of the experimental conditions (*beta* = 75.26, *p* < 0.001, 95% CI [41.43; 109.10]) with participants reporting in average longer temporal estimations in the **BASELINE** (mean = 445.35 ± 218.63) compared to the **OPERANT** condition (mean = 436.36 ± 200.82). However, this difference was no longer significant after Hölm Bonferroni corrections for multiple comparisons (*p* = 0.51). Finally, the analysis on temporal estimations revealed a significant interaction between experimental conditions and interval durations (*F* (4, 819) = 13.20, *p* < 0.001). Participants reported shorter temporal duration in the **OPERANT** condition for the 500 (*beta* =  − 120.92, *p* < 0.001, 95% CI [− 168.77; − 73.06]), 700 (*beta* =  − 118.55, *p* < 0.001, 95% CI [− 166.40; − 70.70]), and 900 ms (*beta* =  − 126.97, *p* < 0.001, 95% CI [− 174.82; − 79.12]) interval durations.

**Figure 3:**
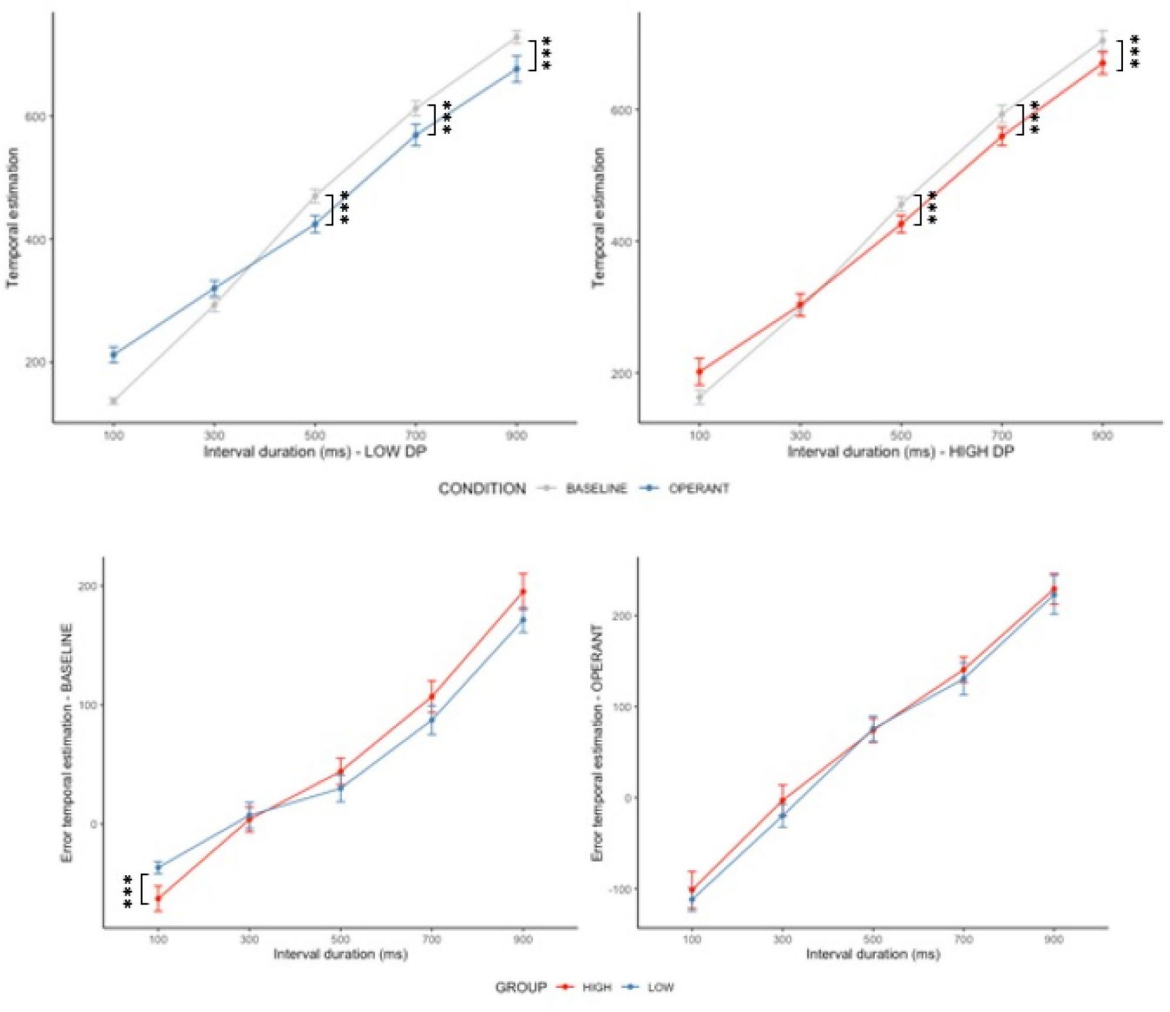
Graphical plot of temporal estimations (top row) and error in temporal estimations (bottom row) for HIGH (red) and LOW (blue) DP for **BASELINE** and **OPERANT** conditions. The bars represent standard errors and *p*-values are reported with *p* < 0.050*, *p* < 0.010** and *p* < 0.001***.

There were no direct effects of DP groups on temporal estimations (*beta* = 26.07, *p* = 0.222, 95% CI [− 16.09; 68.24]), but there was an interaction with the 900 ms interval duration (*beta* =  − 49.74, *p* = 0.043, 95% CI [− 97.85; − 1.62]). Exploratory analyses revealed that this effect was driven by significant differences in temporal distortions (i.e., relative distance between interval duration and temporal estimations) between **HIGH** (100.71 ± 87.60) and **LOW** DP (85.50 ± 73.65) in the **BASELINE** condition (*t* (463) = 2.02, *p* = 0.044, 95% CI [0.44; 29.97]). See Fig. 3 for a graphical representation and Table B in Supplementary Materials for descriptive statistics.

**Figure 4:**
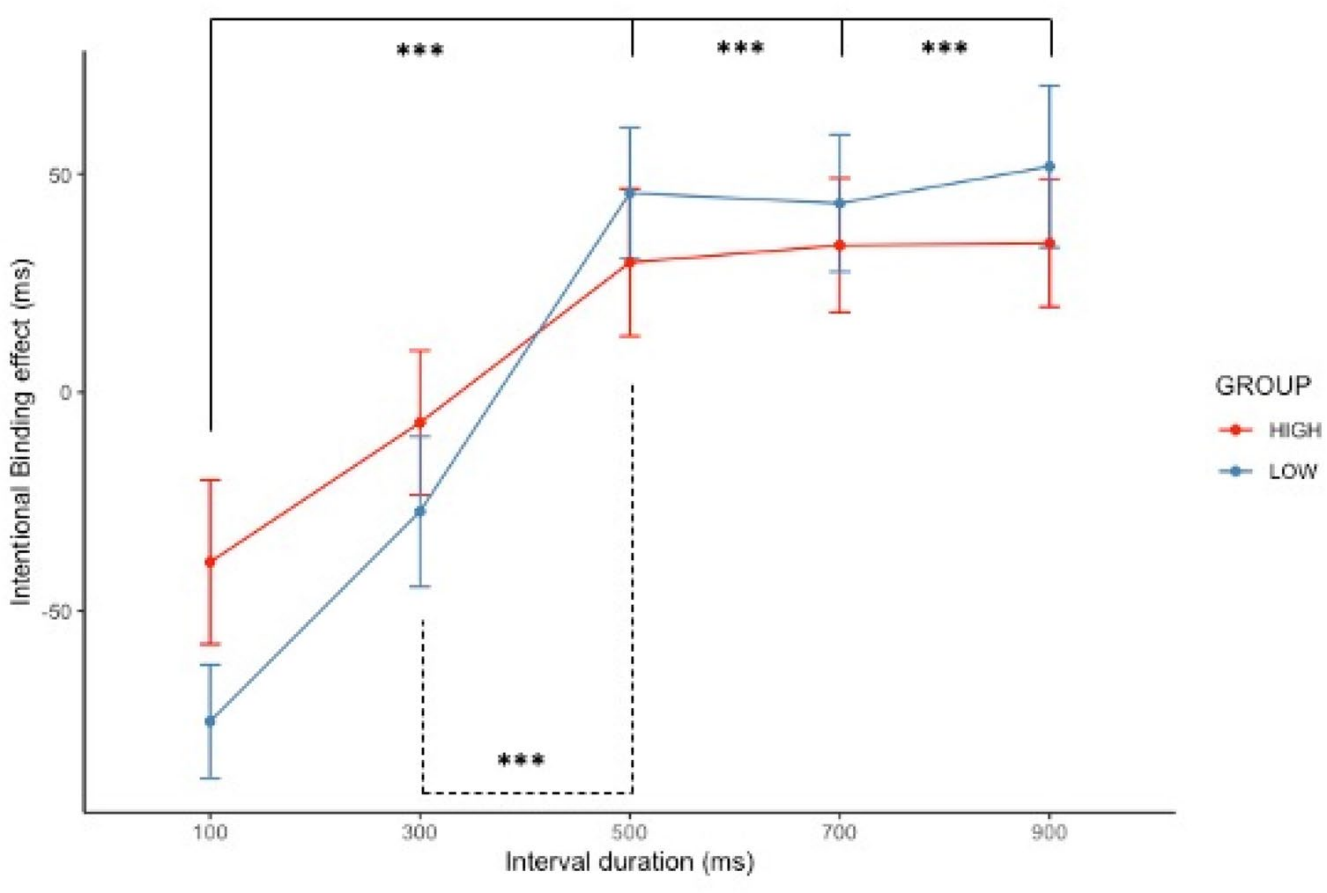
Graphical plot of intentional binding for **LOW** (blue) and **HIGH** DP (red). The bars represent standard errors and *p*-values are reported with *p* < .050*, *p* < .010** and *p* < .001***.

A significant main effect of interval durations was observed on IB effect (*F* (4, 364) = 23.05, *p* < 0.001 and *R*^2^ = 0.43). Post-hoc comparisons showed that participants displayed IB effect for 500 (*beta* = 68.56, *p* < 0.001, 95% CI [31.88; 105.24]), 700 (*beta* = 72.48, *p* < 0.001, 95% CI [35.79; 109.67]), and 900 ms interval durations (*beta* = 72.99, *p* < 0.001, 95% CI [36.31; 109.67]). A significant main effect of DP groups was also observed (*beta* =  − 55.71, *p* = 0.034, 95% CI [− 107.02; − 4.41]) in IB effect (Estimations difference between **BASELINE** and **OPERANT** conditions). The **HIGH** DP group (mean = 10.38 ± 114.59) displayed overall higher intentional binding than the **LOW** DP group (mean = 7.63 ± 119.76). However, this difference was no longer significant after Holm Bonferroni corrections (*p* = 0.80). There was indeed no significant difference between **HIGH** and **LOW** DP groups (*F* (1, 89) = 2.93, *p* = 0.090 and *R*^2^ = 0.89) when considering the overall coefficient slopes of intentional binding, but an effect of participants’ age was found (*beta* = 10.23, *p* < 0.001, 95% CI [4.49; 15.98]). Nevertheless, the DP groups displayed a significant interaction with the 900 ms interval durations (*beta* = 53.98, *p* = 0.040, 95% CI [2.39; 105.57]). Post-hoc comparisons revealed that participants from the **HIGH** DP group displayed significant differences in intentional binding in the 100 ms compared to the 500, 700, and 900 ms interval duration (all *ps* < 0.050). In contrast, participants from the **LOW** DP group displayed significant differences in intentional binding in the 100 ms and 300 ms compared to the 500, 700, and 900 ms interval duration (all *ps* < 0.010). Exploratory analyses on group differences across interval durations failed to reveal any significant difference in IB effect (all *ps* > 0.050), despite a trend for a significant difference for the 100 ms interval duration (*t* (80) = 1.60, *p* = 0.114, 95% CI [− 8.99; 81.91]). See Fig. 4 for a graphical representation and Table C for the descriptive statistics.

### Associations between implicit and explicit sense of agency, CDS facets and SST scales

There was a positive correlation between coefficient slopes associated with explicit and implicit agency (*r* = 0.23, *p* = 0.025, 95% CI [0.03; 0.42]). However, this association displayed a trend for significance in the **LOW** DP group (*r* = 0.25, *p* = 0.096, 95% CI [− 0.04; 0.50]), but not in the **HIGH** DP group (*r* = 0.15, *p* = 0.323, 95% CI [− 0.15; 0.42]).

The CDS facet did not predict any significant changes in coefficient slopes of explicit and implicit sense of agency apart from the CDS facet “Alienation from Surroundings” which predicted steeper coefficient slopes in explicit sense of agency (* R*^2^ = 0.88, *beta* = 0.17, *p* = 0.034, 95% CI [0.01; 0.32]).

The SST item 5 associated with prospective time travel was positively correlated with explicit sense of agency (*rho* = 0.40, *p* = 0.001). In contrast, feelings of estrangement of the self in space (SST items 6, 10 and 14) were negatively correlated with explicit (*rho* =  − 0.35, *p* = 0.001 and *rho* =  − 0.21, *p* = 0.042 for items 6 and 10) and implicit sense of agency (*rho* =  − 0.27, *p* = 0.036, for item 14).

## Discussion

Our study used the IB paradigm to investigate the relationship between DP experiences and the sense of agency in a non-clinical sample. Against our initial hypothesis, no significant differences were found between individuals with low and high occurrences of DP experiences, both for explicit and implicit sense of agency. However, a trend for participants with higher occurrences of DP experiences to display a more time-sensitive explicit sense of agency was observed, potentially driven by the CDS facet “Alienation from Surroundings”. More importantly, significant differences were observed between people with high and low DP experiences when considering time perception.These findings are discussed in detail below.

### Explicit sense of agency

Our study first revealed that both HIGH and LOW DP groups displayed a decreased tendency to experience a sense of agency with increasing duration. This pattern is aligned with Imaizumi & Tanno’s^53^ previous work reporting a similar decrease of the sense of agency with time.

Although group differences did not reach significance, the results indicate a trend for a diminished sense of agency for participants with higher occurrences of DP experiences. Therefore, in our non-clinical population, the distribution did not allow the observation of statistical differences.

While there was no difference between HIGH and LOW DP groups on the explicit sense of agency, the two groups displayed distinct patterns when interval durations are considered. Participants scoring high on the CDS displayed a more time-sensitive explicit sense of agency. Interestingly, this association seems to be driven by the CDS facet ‘Alienation from Surroundings’ that was associated with steeper coefficient slopes of explicit sense of agency.

The lack of association with the other CDS facets suggests that the sense of agency may be less affected by alterations in body awareness (i.e., “mineness”^1^), emotional blunting, or distortion in autobiographical memory alone, but only when accompanied by a sense of detachment from the world. Consequently, the subjective experience of the sense of agency cannot be reduced to body ownership only, but it is intrinsically entangled with the experience of a body acting in the world.

These results suggest a crucial role of “presence” in the external world, understood as “being here”, in order to feel real. One needs to successfully keep track of the reality of the world in order to be able to keep track of one’s self *in* the world. Dynamic interactions with the world are indeed key for self / other differentiation and blurred body / world boundaries have been found to correlate with DP experiences^62^. The importance of active engagements with the environment as an effective way to increase the sense of agency in DP individuals, to “put them back” in their bodies and in the world has been outlined^63^. This view is compatible with previous work suggesting an association between bodily movements, the sense of agency, and the sense of presence^63–65^.

Interestingly, while self-reports of explicit sense of agency decreased with interval duration^53,66,67^ comparisons between the HIGH and LOW DP groups revealed a more nuanced picture. Specifically, the explicit sense of agency decreases linearly (i.e., significantly across interval duration) in the HIGH DP group, whilst the experience of agency of the LOW DP group was less sensitive to interval duration. The evolution of explicit agency with time in the HIGH DP group replicated the pattern observed in Imaizumi & Tanno’s study^53^ that controlled for the sense of self using the Embodied Sense of Self Scale^68^, developed from CDS items. Consequently, these common findings call for further investigations combining both scales for delineating their distinctive contributions in the sense of agency in DP.

### Implicit sense of agency

The implicit measure of agency through IB in relation to DP revealed a more complex pattern.

Although there was a trend for an association between the DP groups and the intentional binding effect, this association seems to be driven by group differences in temporal estimations. In depth examination revealed that individuals with high DP occurrences displayed greater temporal distortion (i.e., over-estimation of short interval and under-estimation of longer interval) in the Baseline condition. Importantly, this difference was *not* significant in the Operant condition. This suggests that performing a voluntary action decreased the difference in temporal estimations between high and low DP, influencing the calculation of the IB effect. This result points to a potentially beneficial effect of movement on people’s sense of self and agency. Interestingly, this observation fits nicely with subjective reports of coping mechanisms associated with DP reported by Ciaunica et al.^63^ suggesting that active engagements with the environment may help individuals to “maintain connection with the world” and share the same temporality as others:^69,70^ “When the Depersonalisation is very deep, (…) it feels like that constant source of interaction is the only thing that allows me to maintain a connection with the world. I’ll also seek physical contact with whoever I’m with”^63^. These findings are thus aligned with a recent online study showing that dance/movement therapy can reduce symptoms of depersonalisation, calling for further investigations^71^.

When considering self-reports of temporal perception, there was a significant and positive association between explicit sense of agency and tendencies for temporal projection in the future. In contrast, self-reports associated with feelings of estrangement of the self (items 6 and 14 from the SST) were negatively associated with explicit and implicit sense of agency, possibly capturing the phenomenological experiences of depersonalisation and its association with the sense of agency. Altogether, these results suggest that there is no direct disruption between DP and the sense of agency but, similarly to schizophrenia, a possible association with altered experience of time^47–49^. Importantly, differences between High and Low DP occurred especially in the absence of voluntary movement, suggesting that movement allows individuals experiencing DP to perceive time similarly to individuals without DP experiences. Although the present study was conducted in a non-clinical population, calling for replications, the obtained results pave the way for further investigation on time perception in depersonalisation in a clinical sample.

### Correlation between implicit and explicit sense of agency

Similarly to Imaizumi & Tanno’s results^53^, the implicit and explicit sense of agency were significantly associated, but this pattern was observed only for the Low DP group, suggesting a discrepancy between explicit and implicit sense of agency in the High DP group. Although typically couched as “losing” one’s sense of self or sense of agency, depersonalisation symptoms, may be linked, on the contrary, to an inability to attenuate self-related inputs and hence to ‘forget’ the self in the background. Alterations in the ability to attenuate self-related information may further increase reflexivity or ‘hyper-reflexivity’, which may explain the discrepancy between the implicit and explicit sense of agency in high DP individuals^72,73^.

However, the association between implicit and explicit sense of agency needs to be taken with caution considering the nonlinear association between intentional binding and temporal delays.

In our study, the expected temporal compression characterizing the intentional binding effect occurred only for interval durations superior or equal to 500 ms. This pattern did not replicate Imaizumi and Tanno’s results^53^ of a decrease of IB with time intervals, which is also usually observed in IB studies (e.g. ^36,67^,). Nevertheless, other studies have suggested that the intentional binding might follow an inverted U-shape, increasing for intervals shorter than 400 ms and decreasing for longer intervals^32,74,75^, although this threshold varies across studies and methods^76^. Interestingly, the temporal duration of 500 ms (or 2 Hz) has already been identified as the preferential rate for finger tapping^77^ and phase transition in motor coordination and neural activation^78,79^. Hence, our findings contribute to further understanding the role of temporal delays on the IB effect, which remains an open question in the literature^32,74,80^.

Taken together, the main findings of this study show that depersonalisation traits are not directly associated with changes in the implicit and explicit sense of agency, but they seem to be interval-dependent, calling for further investigations of the role of depersonalisation on time perception. Indeed, complementary exploratory analysis revealed a significant negative correlation between depersonalisation experiences and future orientation in time measured with the SST, with the High DP group reporting lower propensity to project oneself enthusiastically into the future (see Supplementary Material II, Table A). Interestingly, this self-reported measure modulated the explicit sense of agency and suggests a possible association of time travel (i.e., autonoetic awareness) with depersonalisation facets, a phenomenon already observed in schizophrenia^81^, opening the way for future studies exploring its association with the sense of agency.

Altogether, these results suggest that there may not be a direct disruption between DP and sense of agency per se. Rather, atypical sense of agency in DP may be linked to alterations in time perception in relation to the feelings of being disconnected or alienated from the world. Importantly, differences between High and Low DP groups occurred especially in the absence of movement (Baseline condition), suggesting that moving in the world allows individuals experiencing DP to experience time similarly to typical individuals. Although the present study was conducted in the non-clinical population, it sets the ground for further investigation on the relationship between time perception and feelings of reality or presence in the world both in clinical and non clinical populations. Overall, our findings call for further investigations of the role of time perception in constructing the experience of being a self in the world and its association with intentional binding measures in particular, and with the sense of agency more broadly.

### Limitations and outlook

Our findings yield several important limitations. First, our study was conducted online with limited control, and hence our results must be taken with caution. However, whilst potential artifacts due to technical differences between High and Low DP groups cannot be ruled out, the intentional binding was measured using a within-subject design, mitigating these issues^82^. Second, the mismatch between High and Low DP groups for demographics (i.e., age and gender) also calls for a careful interpretation of the present findings.

The high variability observed in our study underlines possible confounding variables such as demographics or distinct influences of CDS facets that need to be addressed by future studies. Third, the lack of replication of Imaizumi & Tanno’s results^53^ questions the impact of experimental design on measures of the intentional binding effect. Previous studies reported inconsistent findings, suggesting U-shape variations of IB with interval durations^32,74,75^; but this threshold varies across studies, stressing the role of temporal estimations methods^76^. Hence, the discrepancy with the results found in Imaizumi & Tanno^53^ can be attributed to methodological differences such as different intentional binding indexes, self-reports of temporal estimations and the counterbalanced order of conditions. Furthermore, whilst Imaizumi & Tanno’s study was conducted in a Japanese population, the present study was conducted in a French speaking population. Hence the role of cultural differences cannot be ruled out and require further studies investigating the sense of agency cross culturally^55,82^. Finally, the current debates on the nature of the processes measured by the intentional binding task requires further investigations^30,37^. Whilst some authors raised the question of “intention” in the intentional binding effect^40–42^; other suggested that the IB reflects a specific subcomponent of an overall “temporal binding” effect, resulting from the perception of causal relations between intentional actions and their effects^33,40,83,84^—see also more recently^37^. Consequently, further investigations are required to disentangle potential procedural confounds and to delineate distinct temporal windows of integration across various sensory modalities.

## Conclusion

Our study provides novel insights on the association of DP experiences occurrences on explicit and implicit sense of agency, measured through the intentional binding task. Contrary to other conditions associated with altered sense of self such as schizophrenia, our study did not reveal significant differences between individuals reporting high and low DP experiences on overall indices of explicit and implicit sense of agency. However, our results suggest specific associations between DP experiences and distorted experience of time.

First, people with high DP experiences displayed a trend for a more time-sensitive sense of explicit agency, that seems to be especially driven by the facet “*Alienation from Surroundings*”, characterized by experiences of disconnection from reality and external world associated with derealisation, a common symptom of depersonalisation. Therefore, this first result underlines the role of “presence” in the experience of agency, paving the way for further investigations on its possible pivotal role on moderating the sense of agency in depersonalisation.

Second, our study revealed a nonlinear association between intentional binding and interval duration. Although beyond the initial scope of our study, this result calls for further investigations of the role of temporal estimation in the intentional binding task. Importantly, whilst depersonalisation seems to be associated with lower tendencies for intentional binding, group differences were only observed for short intervals and in the absence of intentional action. These results pave the way for investigating the role of self-initiated movements in reducing DP symptoms.

Finally, whilst implicit and explicit sense of agency are correlated for individuals with low DP, these measures were dissociated for individuals with high occurrences of DP experiences. This third finding calls for further investigations in clinical populations, to disentangle the mechanisms associated with explicit and implicit sense of agency, which may use active engagement with the world as a therapeutic intervention to help people with DP to get back into the world, and hence into their body and self.

### Supplementary Information

Supplementary Information.

## Data Availability

The dataset generated by this study is available in the Open Science Framework repository: https://osf.io/vf9hw/?view_only=513a8c23ad4145418ea7f5dffad526f1.
