# Supplementary material for: Explicit and implicit sense of agency in depersonalisation experiences
Source: Sci Rep. 2024 Jul 4;14:15396. doi: 10.1038/s41598-024-65862-z (PMC11224402; doi:10.1038/s41598-024-65862-z)
Supplement: Supplementary file 1 — Supplementary Information. [file 41598_2024_65862_MOESM1_ESM.docx]

**SUPPLEMENTARY MATERIAL**

|  | **Explicit sense of agency** | | | | |
| --- | --- | --- | --- | --- | --- |
|  | **100 ms**  5.60 ± 2.08 | **300 ms**  4.99 ± 1.81 | **500 ms**  4.42 ± 1.77 | **700 ms**  4.02 ± 1.82 | **900 ms**  3.72 ± 1.98 |
| **LOW DP** | 5.38 ± 2.24 | 4.88 ± 2.07 | 4.52 ± 2.04 | 4.16 ± 2.08 | 3.99 ± 2.31 |
| **HIGH DP** | 5.83 ± 1.90 | 5.08 ± 1.52 | 4.32 ± 1.45 | 3.86 ± 1.51 | 3.45 ± 1.56 |

**Table A:** Descriptive statistics for explicit sense of agency for each interval duration

|  | **Temporal estimation** | | | | | | | | | |
| --- | --- | --- | --- | --- | --- | --- | --- | --- | --- | --- |
|  | **BASELINE CONDITION** | | | | | **OPERANT CONDITION** | | | | |
|  | **100 ms**  149.47  (57.39) | **300 ms**  294.44  (72.88) | **500 ms**  463.00  (75.67) | **700 ms**  602.99  (86.59) | **900 ms**  716.86  (88.25) | **100 ms**  206.70 (114.94) | **300 ms**  311.63 (100.94) | **500 ms**  425.21 (91.64) | **700 ms**  564.46 (108.09) | **900 ms**  673.82  (130.99) |
| **LOW** | 136.60  (35.42) | 292.74 (75.28) | 470.04 (76.84) | 612.70 (82.51) | 728.59  (71.81) | 211.86  (88.22) | 319.98 (85.18) | 424.39 (95.15) | 569.41 (121.10) | 676.88 (147.46) |
| **HIGH** | 162.62  (71.43) | 296.19  (71.13) | 455.80  (74.60) | 593.06  (90.38) | 704.87 (101.78) | 201.43  (137.83) | 303.09  (115.18) | 426.05  (88.94) | 559.40  (94.04) | 670.69 (113.28) |

**Table B:** Descriptive statistics of temporal estimation from participants with **LOW DP** and **HIGH DP** experimental groups for interval durations and for **BASELINE** and **OPERANT** conditions with standard deviations reported in parentheses.

|  | **Intentional binding (BASELINE - OPERANT)** | | | | |
| --- | --- | --- | --- | --- | --- |
|  | **100 ms**  -57.23 ± 110.64 | **300 ms**  - 17.18 ± 114.61 | **500 ms**  37.79 ± 108.02 | **700 ms**  38.53 ± 105.50 | **900 ms**  43.04 ± 113.92 |
| **LOW DP** | -75.26 ± 89.03 | -27.25 ± 117.62 | 45.65 ± 102.25 | 43.29 ± 107.37 | 51.71 ± 127.21 |
| **HIGH DP** | -38.80 ± 127.43 | -6.90 ± 111.80 | 29.76 ± 114.18 | 33.66 ± 104.51 | 34.19 ± 99.15 |

**Table C:** Descriptive statistics of intentional binding from **LOW** and **HIGH** DP groups for the interval durations.

| **SST items** | **HIGH** | **LOW** | **Non-parametric T-tests** |
| --- | --- | --- | --- |
| **1. J’ai le sentiment (je sens) que le temps passe plus vite que d’habitude** | 4.04 ± 1.05 | 2.34 ± 1.05 | *W* = 1867, *p* < .001 |
| **2. J’ai le sentiment (je sens) que le temps passe moins vite que d’habitude** | 2.04 ± 1.19 | 1.68 ± 0.81 | *W* = 1233, *p* = .210 |
| **3. J’ai le sentiment de vivre plutôt dans le présent, dans l’ici et maintenant** | 3.33 ± 1.21 | 3.68 ± 1.09 | *W* = 896.5, *p* = .0141 |
| **4. J’ai le sentiment que je pense souvent à des événements du passé** | 3.43 ± 1.44 | 3.30 ± 1.23 | *W* = 1172, *p* = .474 |
| **5. J’ai le sentiment que je me projette avec enthousiasme dans l’avenir** | 3.30 ± 1.15 | 3.81 ± 1.19 | *W* = 822, *p* = .041 |
| **6. J'ai le sentiment, lorsque je me regarde dans un miroir, que mon visage me semble étranger, non-familier** | 2.20 ± 1.07 | 1.04 ± 0.20 | *W* = 1803, *p* < .001 |
| **7. J’ai le sentiment que le son de ma propre voix quand je parle est souvent étranger, non-familier** | 2.15 ± 1.09 | 1.13 ± 0.54 | *W* = 1752, *p* < .001 |
| **8. J’ai le sentiment, quand je l’écoute enregistrée sur le répondeur, que le son de ma propre voix est souvent étranger, non-familier** | 3.67 ± 1.23 | 1.98 ± 1.17 | *W* = 1784, *p* < .001 |
| **9. J’ai le sentiment, quand ils me parlent, que le son de la voix de mes proches est souvent étranger, non-familier** | 1.37 ± 0.74 | 1.00 ± 0.00 | *W* = 1339.5, *p* < .001 |
| **10. J’ai le sentiment, quand je l’écoute enregistrée sur un répondeur, que le son de la voix de mes proches est souvent étranger, non-familier** | 1.67 ± 0.79 | 1.04 ± 0.20 | *W* = 1582.5, *p* < .001 |
| **11. J’ai le sentiment, lorsqu’ils me parlent, que le son de la voix des inconnus est irritante.** | 2.37 ± 1.14 | 1.11 ± 0.31 | *W* = 1770.5, *p* < .001 |
| **12. J’ai le sentiment que le son des bruits familiers de la vie de tous les jours (brossage des dents, le bruit de l’eau du robinet qui coule, le bruit de mes pas sur le plancher, etc.) est trop fort et irritant** | 2.57 ± 1.34 | 1.06 ± 0.32 | *W* = 1800.5, *p* < .001 |
| **13. J’ai le sentiment que les objets familiers de la vie de tous les jours (table, chaise, lavabo, etc.) sont plus grands et plus proches de mon corps que d’habitude** | 1.33 ± 0.79 | 1.00 ± 0.00 | *W* = 1269, *p* = .003 |
| **14. J’ai le sentiment que les objets familiers de la vie de tous les jours (table, chaise, lavabo, etc.) sont plus petits et plus éloignés de mon corps que d’habitude** | 1.30 ± 0.70 | 1.00 ± 0.00 | *W* = 1292.5, *p* = .002 |

**Table D:** Descriptive statistics and comparisons between HIGH and LOW DP for SST items.
